# Crude Oil-Degradation and Plasmid Profile of Nitrifying Bacteria Isolated from Oil-Impacted Mangrove Sediment in the Niger Delta of Nigeria

**DOI:** 10.1007/s00128-012-0609-8

**Published:** 2012-03-30

**Authors:** R. C. John, G. C. Okpokwasili

**Affiliations:** 1 Department of Microbiology, University of Port Harcourt, Port Harcourt, Rivers State Nigeria; 2 University of Uyo, Uyo, Nigeria

**Keywords:** Biodegradation, Crude oil, Nitrifying bacteria, Mangrove sediment

## Abstract

The crude oil degradability and plasmid profile of autotrophic nitrifying bacteria, *Nitrosomonas* and *Nitrobacter* species, isolated from mangrove sediment in the Niger Delta of Nigeria were studied. The effects of temperature, pH and optical density on the utilization of different carbon sources by the bacteria were also investigated. Results showed that nitrifying bacteria could utilize kerosene, diesel oil, jet fuel and engine oil as carbon sources. None utilized hexane and xylene but moderate growth was observed in benzene, phenol and toluene. However, their ability to utilized crude oil varied both in rates of utilization and in growth profiles. Mixed culture of the isolates degrades 52 % of crude oil introduced into the medium followed by *Nitrosomonas* sp. with 40 % degradation. The least was *Nitrobacter* sp. with 20 % degradation. The ability of the autotrophs to degrade crude oil was found to be plasmid-mediated through curing experiment and electrophoresis. The size of the plasmid involved was estimated to be 23 kb. The high crude oil utilization of the mixed culture implies that nitrifying bacteria isolated from contaminated ecosystem are excellent crude oil degraders and can be harnessed for bioremediation purposes.

Microbiological activity is affected by many environmental factors including energy source, donors and acceptors of electrons, nutrients, pH, and temperature. These parameters influence how quickly microorganisms adapt to the environment (Vieira et al. 2007). Hydrocarbon degradation by microbial population in natural environment is influenced by physical, chemical and biological factors that contribute to the degradation of petroleum and individual hydrocarbons. Rate of biodegradation depends greatly on the composition, state, and concentration of the oil or hydrocarbons, with dispersion and emulsification enhancing rates in aquatic systems and absorption by soil particulates being the key feature of terrestrial ecosystems. Salinity and pressure may also affect biodegradation rates in some aquatic environments while moisture and pH may limit biodegradation in soils (Vieira et al. 2007). Adaptation by prior exposure of microbial communities to hydrocarbons also may increase hydrocarbon degradation rates (Leahy and Colwell 1990). Lindstorm et al. (1991) have reported enhanced growth of hydrocarbon degrading microbes in oil spilled site after the application of fertilizer. Yeasts strain identified as *Candida tropicalis* strains 7y and 15y have also been reported to be efficient oil degraders (Palittapongarnpim et al. 1998).

Certain plasmids play important role in the adaptation of natural microbial populations to oil and other hydrocarbons (Okpokwasili et al. 1986). Some degradation, including the breakdown of C5 to C12 n-alkanes, naphthalene and toluene pathways have been extensively characterized and are generally located on large catabolic plasmids (Gary et al. 1990). Similarly, several environmental isolates of *Acinetobacter* sp. and *Alcaligenes* sp. (Lal and Khanna 1996), *Arthrobacter* sp. (Efroymson and Alexander 1991) and two *Rhodococcus* strains (Malachowsky et al. 1994) have been found to degrade both alkanes and naphthalene, although the genes and catabolic pathways responsible were not described.

The Niger Delta ecosystem is subjected to man-induced changes and seriously threatened by increasing environmental deterioration. The aquatic ecosystem of the region faces increasing ecological and toxicological problems from the release of petroleum pollutants (Mayerson et al. 1981). Hydrocarbon utilizing microorganisms; *Micrococcus, Pseudomonas, Bacillus, Aeromonas, Serratia, Proteus, Penicillium, Aspergillus, Candida* and *Geotrichum* have been isolated by enrichment techniques from sediment and water samples collected from oil spill sites in the Niger Delta of Nigeria (Benka-Coker and Ekundayo 1997). The same researchers observed quantitative changes in the oil content due to microbial degradative activities. Degradation was more pronounced with mixed cultures than with single cultures of the microorganisms. Similarly, the relative capabilities of two bacterial isolates *Serratia marcescens* OCS-21 and *Acinetobacter calcoaceticus* COU-27 and yeast isolate; *Candida tropicalis* PFS-95 in degrading Trans Niger pipeline crude oil has been also reported by Ijah (1998). The yeast isolate showed more proficiency in degrading the crude oil. Components of length C_12_ to C_32_ were extensively degraded by the yeast after 16 days of incubation.

Several methods have been introduced to increase the rate of biodegradation in the soil and they include oxygenation by excavation of the soil, nutrient supplementation and microbial seeding (Atlas and Bartha 1992). The reduced ability of nitrifying bacteria to participate in remediation of oil-contaminated sediment has been a limiting factor. However, Deni and Penninck (1999) have reported that nitrifying bacteria in polluted soil initiate a syntrophic pathway that provides intermediates for heterotrophic bacterial activity and thus are excellent candidates for hydrocarbon remediation. In this work, the crude oil-degradability and plasmid profile of nitrifying bacteria from oil-impacted mangrove sediment in the Niger Delta of Nigeria was investigated.

## Materials and Methods

The hydrocarbons such as benzene, hexane, xylene, phenol and toluene used in this study were BDH Laboratory supplies from England. Kerosene, diesel and engine oil were purchased from petroleum marketers in Nigeria, while crude oil was obtained from Exxon Mobil Producing Nigeria Unlimited, Eket, Nigeria.

The test organisms were isolated from oil-contaminated mangrove sediment in Iko, Akwa Ibom State, Nigeria. The intertidal sediment samples (0–15 cm depth) were collected using short core sampler and transferred into sterile polyethylene bags and then transported to the laboratory for analysis. Precisely 5 g of the sediment was suspended in 45 ml of sterile phosphate buffer containing 139 mg of K_2_HPO_4_ and 27 mg KH_2_PO_4_ per litre (pH 7.0) and shaken at 100 rpm for 2 h (Deni and Penninck 1999). Sub-samples of the suspensions were diluted in sterile microtitre plates containing Winogradsky media for isolation of *Nitrobacter* and *Nitrosomonas* sp. (Colwell and Zambruski 1972). *Nitrosomonas* sp. was isolated using Winogradsky medium for nitrification Phase I which consists of (NH_4_)_2_SO_4_, 2.0 g; K_2_HPO_4_, 1 g; MgSO_4_·7H_2_O, 0.5 g; NaCl, 2.0 g; FeSO_4_·7H_2_O, 0.4 g; CaCO_3_, 0.01 g, Agar, 15.0 g and distilled water, 1,000 ml. On the other hand *Nitrobacter* was isolated using Winogradsky medium for nitrification Phase II which consists of KNO_2_, 0.1 g; Na_2_CO_3_, 1 g; NaCl 0.5 g; FeSO_4_·7H_2_O, 0.4 g; Agar, 5.0 g and distilled water, 1,000 ml. The media were dispersed into sterile Petri dishes after cooling to about 45°C. The Petri dishes were then inoculated and incubated aerobically for 7 days at room temperature (28 ± 2°C) for *Nitrosomonas* and *Nitrobacter* sp. Further identification and characterization of pure cultures of isolates were undertaken using the criteria of Holt et al. (1994).

Pure isolates obtained were cultured on mineral basal medium supplemented with 1 % v/v crude oil. The mineral basal medium was composed of (NH_4_)_2_SO_4_, 1.0 g; KH_2_PO_4_, 1.0 g; K_2_HPO_4_, 1.0 g; MgSO_4_, 0.2 g; CaCl_2_, 0.02 g; FeCl_3_·6H_2_O; 0.004 g and brackish water (mixture of salt and fresh water) 30ppt-1,000 ml at pH-7 ± 0.2. Microbial growth and production of zone of clearing around the microbial colonies on medium were regarded as evidence of ability to utilize oil. This was graded as strong (+++), moderate (++), weak (+) and no (−) degrading potential. This procedure has earlier been adopted by Ekundayo and Obire (1987).

The test cultures were separately grown in 45 ml nutrient broth and incubated at 37°C for 3 days. The flasks were agitated on a shaker and the cultures were harvested when fully grown and suspended in 10 ml phosphate buffer (50 mM, pH 7.2) and then centrifuged at 4°C for 10 min at 10,000×*g*. Thereafter, 2 g each of the isolates were standardized in 5 ml saline water by spectrophotometry to achieve uniform population of the mixed cultures of nitrifying bacteria. This was resuspended in10 ml of mineral salt medium to a final population of about 10^4^ cfu per ml of the isolates and used as starter culture for the degradation experiment.

In addition to growth on crude oil, the purified strains were also tested for growth on other petroleum products. These include diesel oil, kerosene, engine oil and jet fuel. Other compounds tested were benzene, toluene, xylene, hexane, and phenol. Mineral basal medium containing 1 % v/v of the above product was inoculated and incubated for 14 days at 28°C. Cultures without increase in turbidity over initial optical density and non-inoculated control were scored as no growth (−) while cultures with increased turbidity significantly greater than the control (i.e. growth attenuation, optical density OD reading above 0.2.) were scored as growth (+).

The degradation rates of bacterial isolates were determined using crude oil supplemented medium. Precisely 2 ml of 48 h old broth cultures of each organism were introduced into 24 sterile 250 ml capacity Erlenmeyer flasks containing 200 ml of sterile mineral basal medium supplemented with 2 ml of crude oil as source of carbon and agitated at 180 rpm at 28 ± 0.2°C for 35 days on a rotary shaker. A set of 6 flasks with mineral basal medium and 2 ml of crude oil but without test organism served as control. During incubation, representative samples from the four sets of flasks were withdrawn at interval of 5, 10, 15, 20, 25, 30 and 35 days using destructive approach. The temperature, pH, optical densities (OD) of the medium in different flasks were measured and the total viable cells of the organisms were also determined by spread plate count. Counts for the test organisms were recorded while the residual crude oil was determined gravimetrically using diethyl ether as the extraction solvent. This method has earlier been adopted by many authors (Odu 1972; Ijah and Ukpe 1992; Itah and Essien 2005). For each sample, 5 ml diethyl ether was vigorously shaken manually. The mixtures were then separated using a laboratory funnel and evaporated at room temperature to remove the solvent thus leaving the residual crude oil. The weight of the crude oil residues were then determined using a standard curve. The percentage biodegradation of the crude oil was determined as follows:

$$ \% \,{\text{degradation}} = \frac{{{\text{a}} - {\text{b}}}}{{\text{a}}} \times \frac{{{\text{100}}}}{1} $$

where a is the weight of crude oil control; b is the weight of crude oil remaining in the each case.

Before isolation of plasmid having subsceptible degradation gene, plasmid curing suggested by Hardy (1993); Sheikh et al. (2003); Fujii et al*.* (1997) and Trevors (1986) were performed to show whether the oil degrading gene is plasmid encoded or chromosomal encoded. The bacterial isolates were cultured in broth medium containing the hydrocarbon of preference (in which the bacteria had shown the highest growth). The media were used in a late exponential phase as starter culture in which 0.1 ml of the culture was added to 100 ml of nutrient broth containing ethidium bromide (100 μg/ml) or acridine orange (0.10 mg/ml). This was incubated at 37°C for 24 h. Thereafter, the broth was agitated to homogenize the content and loopful of the broth medium were subcultured on nutrient agar plate and also on MBM agar containing crude oil. The plates were incubated at 37°C for 24 h and the colonies counted. Colonies that failed to grow on MBM agar plates were considered cured.

Antimicrobial susceptibility of bacterial isolates was performed on MBM agar plates using a modified disc diffusion method. In this case 0.1 ml of test isolates was seeded in Petri dishes containing MBM agar and allowed to stand for 30 min to enable the inoculated organisms to pre-diffuse. Thereafter commercial discs containing 10 μg each of Amplicillin, Tetracycline, Streptomycin, Cefadroxil, Amoxillin, 30 μg of Chloramphenicol and 50 μg of Erythromycin were aseptically placed on the surfaces of the sensitivity agar plates with sterile forceps and were incubated at 37°C overnight. After incubation, zones of inhibition were observed against the entire antibiotic used except ampicillin and cefadroxil. The presence of zone of inhibition is indicative of chromosome-mediated resistance (plasmid not cured) while absence of zone of inhibition was indicative of plasmid-mediated resistance (plasmid cured). The cured strains were sub-cultured on medium with the appropriate (original) hydrocarbon to confirm loss of degradative ability.

Plasmid DNA was extracted by alkali treatment method described by Kado and Liu (1981) with slight modification. After electrophoresis on a 0.7 % horizontal agarose gel at 50 V for 3 h, the gels was stained with ethidium bromide and band visualized with a UV transmilluminator (Coral and Karagoz 2005). Molecular-sized plasmids were determined by comparison with Lambda DNA Hind III digest molecular weight marker.

## Results and Discussion

**Fig. 1:**
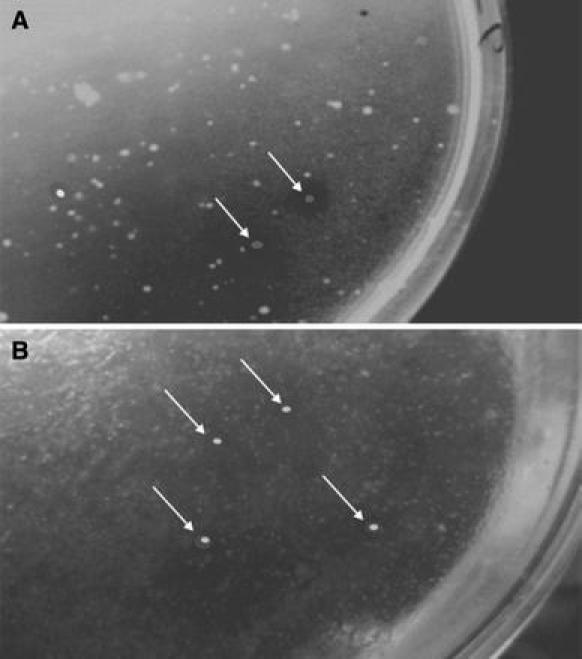
Clear zone (*Arrow*) of crude oil-degrading colonies on MBM agar supplemented with crude oil. **a** *Nitrobacter* sp. **b** *Nitrosomonas* sp.

Many bacterial and fungal isolates from crude oil-polluted soil have been found to degrade crude oil and spent oil. They include *Pseudomonas putida, Pseudomonas aeruginosa, Bacillus subtilis, Alcaligenes eutrophus, Micrococcus luteus, Acinetobacter lwoffi, Proteus* sp*., Aspergillus* sp.*, Penicillium* sp.*, Rhizopus* sp*.* and Candida sp. (Okerentugba and Ezeronye 2003; Nwachukwu et al. 2000; Ilori and Amund 2000; Amund et al. 1993; Okpokwasili and Amanchukwu 1988). However, these isolates had moderate growth on benzene, toluene and phenol but could not degrade hexane and xylene. This has been attributed to the membrane toxicity and non-possession of the necessary enzymes (Igwo-Ezikpo et al. 2006). This study has shown that *Nitrobacter* sp. and *Nitrosomonas* sp. strains isolated from crude oil contaminated mangrove sediment exhibited strong crude oil degradability as indicated by the formation of clearing zones (Fig. 1) and subsequent utilization of crude oil and refined products as sole source of carbon and energy.

Growth profile of the individual and mixed cultures revealed that *Nitrobacter* and *Nitrosomonas* species tested grew well at temperature and pH ranges of 20–28.5°C and 7.0–8.0 respectively. The level of hydrocarbons utilized by the organisms is presented in Table 1. The results indicated that the organisms could utilize crude oil and its petrochemical products, such as diesel, kerosene, jet fuel and engine oil for growth. This agrees with the reports of Deni and Penninck (1999), Okpokwasili and Odokuma (1994) and John et al. (2010, 2011) that nitrifying bacteria is an excellent degrader of crude oil and has ability to utilize crude oil and its products.

**Table 1:** Nitrifying bacterial growth test on crude oil and different hydrocarbons

Substrates	*Nitrobacter* sp.	*Nitrosomonas* sp.	Mix cultures
Crude oil	++	++	++
Kerosene	++	++	++
Diesel	++	++	++
Engine oil	+	++	++
Jet fuel	++	++	++
Benzene	−	+	+
Toluene	−	+	+
Xylene	−	−	−
Hexane	−	−	−
Phenol	−	+	+

Growth was followed by measuring the increase of OD at 600 nm of the culture for 7 days

++ Heavy growth: OD 600 nm > 0.2

+ Moderate growth: OD 600 nm > 0.1

− No growth: OD 600n < 0.02

**Fig. 2:**
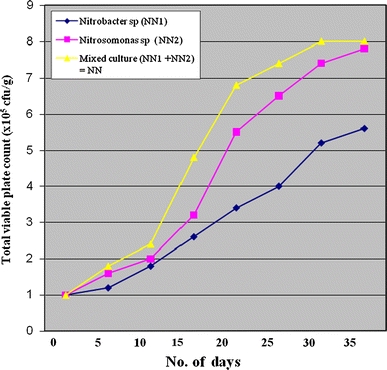
Total viable plate count (×10^5^ cfu/g) of *Nitrobacter*, *Nitrosomonas* and mixed culture

The growth profile of the isolates in the crude oil contaminated soil is presented in Fig. 2. Mixed culture of the two strains showed highest cell density of 8. × 10^5^ cfu/g followed by *Nitrosomonas* sp. with 7.8 × 10^5^ cfu/g. *Nitrobacter* sp. had the least cell density of 5.6 × 10^5^ cfu/g. It was also observed that degradation of crude oil with mixed culture of bacteria was more effective. This may be ascribed to the fact that individual microorganisms may metabolize only a limited range of substrates, while the assemblage of different bacterial strains with broader enzymatic capability has a greater ability to degrade complex mixtures (Mueller et al. 1997; Ellis et al*.* 1991; Trzesicka-Mlynartz and Ward 1995). Their capability to degrade hydrocarbons was further confirmed by the percentage of weight losses of the original level of crude oil (Table 2).

**Table 2:** Weight losses from crude oil resulting from the growth of nitrifying bacteria

Isolates	0	5	10	15	20	25	30	35
Nitrobacter sp.	2 ml	1.90	1.86	1.78	1.74	1.68	1.62	1.6
		(5 %)	(7 %)	(11 %)	(13 %)	(16 %)	(19 %)	(20 %)
Nitrosomonas sp.	2 ml	1.82	1.70	1.65	1.52	1.44	1.30	1.2
		(9 %)	(15 %)	(17.5 %)	(24 %)	(28 %)	(35 %)	(40 %)
Mixed culture	2 ml	1.6	1.46	1.35	1.28	1.18	1.12	0.96
		(20 %)	(27 %)	(32.5 %)	(36 %)	(41 %)	(44 %)	(52 %)
Control	2 ml	1.99	1.99	1.99	1.97	1.97	1.96	1.96
		(0.5 %)	(0.5 %)	(0.5 %)	(1.5 %)	(1.5 %)	(2 %)	(2 %)

Values indicate % weight losses as a measure of degradation rate

However, the degree of weight loss was observed to increase with increase in incubation period but varied with the different microbial species tested. Mixed culture of *Nitrobacter* and *Nitrosomonas* exhibited the highest capability to degrade crude oil with 66.8 % reduction in weight of the crude oil after 35 days of incubation.

The crude oil-degrading capability of *Nitrosomonas* sp. was also remarkable (55.2 %) over the same period of incubation. The least was *Nitrobacter* sp. with 46.4 % crude oil degradability. This is despite the impressive (high) level of attenuance (Table 3) where *Nitrobacter* and *Nitrosomonas* species grew at equal optical densities of 0.6 within 30–35 days of incubation. This suggests that the ability of microorganisms to produce turbidity in culture medium might not necessarily be an indication of its hydrocarbon-degrading capability. The results in Table 3 also revealed that the temperature and pH of the medium during oil degradation for 35 days fluctuated between 27 and 28.5°C and between 7.0 and 8.0 respectively.

**Table 3:** Changes in OD, Temperature and pH of medium during crude oil degradation by single and mixed cultures of *Nitrobacter* and *Nitrosomonas* species

Incubation time (days)	*Nitrobacter* sp.			*Nitrosomonas* sp.			Mixed culture		
	OD	Temp.	pH	OD	Temp.	pH	OD	Temp.	pH
Control	0	28	7.0	0	28	7.0	0	28	7.0
5	0.1	28	8.0	0.2	28	8.0	0.3	28	8.0
10	0.25	27.5	7.8	0.3	28	7.8	0.5	27.5	7.5
15	0.3	28	8.0	0.36	28	8.0	0.6	28	8.0
20	0.5	27.5	7.8	0.42	28	7.5	0.64	28	7.5
25	0.54	28.5	7.8	0.58	28	7.5	0.7	28	7.5
30	0.6	28.5	7.6	0.6	28.5	7.0	0.8	28.5	7.0
35	0.64	28.5	7.6	0.64	28.5	7.0	0.8	28.5	7.0

**Fig. 3:**
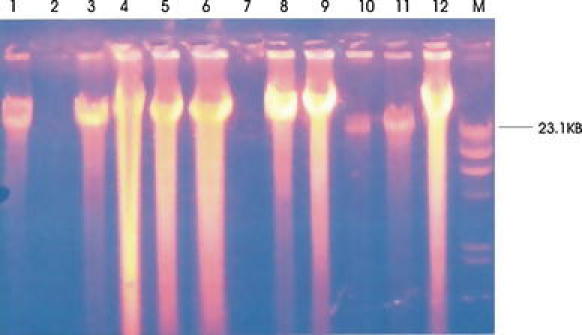
Electrophoretic separation profile of plasmid DNAs from cured and non-cured strains of *Nitrosomonas* and *Nitrobacter* sp. Lines 1, 3, 4, 5 and 6 plasmids from *Nitrosomonas* sp.; Lines 8, 9, 10, 11 and 12 plasmids from *Nitrobacter* sp. Lines 2 and 7 are cured strains of *Nitrosomonas* and *Nitrobacter* sp. respectively M: molecular weight marker (Hind III digest)

Catabolic pathways, which encoded different aromatic hydrocarbon degradation routes, are frequently located on plasmids, although degradative genes can be located on either chromosome or plasmid (Coral and Karagoz 2005). Curing experiments were carried out in order to eventually demonstrate that cured strains were not able to grow in mineral basal medium containing crude oil. It was further verified by carrying out antimicrobial susceptibility of the isolates (Table 4). Our findings show that the abilities to degrade crude oil by *Nitrosomo*nas and *Nitrobacter* species are plasmid-encoded. Agarose gel electrophoretic separation profiles (Fig. 3) of plasmidic DNA isolated from non-cured cultures showed that *Nitrosomonas* and *Nitrobacter* species harboured single plasmids with high molecular weight (23–54 kb). To determine the plasmid harbouring the catabolic genes involved in crude oil degradation, electrophoretic profiles of plasmids isolated from the cured strains were compared with those of the non-cured ones. However, the large (54 kb) plasmids common to both strains in *Nitrosomonas* and *Nitrobacter* species (Line 2 and 7) were cured respectively. These results indicate that catabolic genes responsible for crude oil degradation were located on the plasmid. Hence, nitrifying bacteria can persist in contaminated soil containing limited amount of pollutant for several years (Deni and Penninck 1999).

**Table 4:** Zone of inhibition of different antibiotics against *Nitrobacter* and *Nitrosomonas* species

S/No.	Antibiotics	Abbreviation	*Nitrobacter* sp.	*Nitrosomonas* sp.
			Zone of inhibition	Zone of inhibition
1.	Ampicillin	AP	0	16
2.	Tetracyclin	TC	9	14
3.	Streptomycin	SM	7	9
4.	Cefadroxil	CF	5	0
5.	Amoxillin	AM	4	7
6.	Chloramphenical	CR	8	6
7.	Erythromycin	ET	3	2

This study has revealed a relationship between crude oil degradation and plasmid profile of nitrifying bacteria. It shows that nitrifying bacteria isolated from crude oil-contaminated ecosystem have ability to proliferate and degrade hydrocarbons. It can be deduced that their presence in polluted substrate encourage the development of adaptive features such as plasmid which support hydrocarbon cometabolism. Therefore, the degradative capabilities of the evaluated organisms could be explored in bioremediation campaigns for oil contaminated agricultural soils in the Niger Delta.
